# Insights into the Mechanisms of *Lactobacillus acidophilus* Activity against *Entamoeba histolytica* by Using Thiol Redox Proteomics

**DOI:** 10.3390/antiox11050814

**Published:** 2022-04-22

**Authors:** Lotem Sarid, Eva Zanditenas, Jun Ye, Meirav Trebicz-Geffen, Serge Ankri

**Affiliations:** Department of Molecular Microbiology, Ruth and Bruce Rappaport Faculty of Medicine, Technion, Haifa 31096, Israel; lotemsarid@campus.technion.ac.il (L.S.); zanditenas@campus.technion.ac.il (E.Z.); junye@campus.technion.ac.il (J.Y.); meiravg@technion.ac.il (M.T.-G.)

**Keywords:** *Entamoeba histolytica*, *Lactobacillus acidophilus*, probiotic, redoxomics, cysteine proteases

## Abstract

Amebiasis is an intestinal disease transmitted by the protist parasite, *Entamoeba histolytica*. *Lactobacillus acidophilus* is a common inhabitant of healthy human gut and a probiotic that has antimicrobial properties against a number of pathogenic bacteria, fungi, and parasites. The aim of this study was to investigate the amebicide activity of *L. acidophilus* and its mechanisms. For this purpose, *E. histolytica* and *L. acidophilus* were co-incubated and the parasite’s viability was determined by eosin dye exclusion. The level of ozidized proteins (OXs) in the parasite was determined by resin-assisted capture RAC (OX–RAC). Incubation with *L. acidophilus* for two hours reduced the viability of *E. histolytica* trophozoites by 50%. As a result of the interaction with catalase, an enzyme that degrades hydrogen peroxide (H_2_O_2_) to water and oxygen, this amebicide activity is lost, indicating that it is mediated by H_2_O_2_ produced by *L. acidophilus*. Redox proteomics shows that *L. acidophilus* triggers the oxidation of many essential amebic enzymes such as pyruvate: ferredoxin oxidoreductase, the lectin Gal/GalNAc, and cysteine proteases (CPs). Further, trophozoites of *E. histolytica* incubated with *L. acidophilus* show reduced binding to mammalian cells. These results support *L. acidophilus* as a prophylactic candidate against amebiasis.

## 1. Introduction

Amebiasis is an enormous global medical problem because of poor sanitary conditions and unsafe hygiene practices existing in many parts of the world. According to the World Health Organization, 50 million people in India, Southeast Asia, Africa, and Latin America suffer from amebic dysentery and amebiasis causes the death of at least 100,000 individuals each year. The main mode of transmission for amebiasis is the ingestion of food or water that is contaminated with feces containing *E. histolytica* cysts. After the cyst form has been swallowed by the host, excystation occurs in the intestinal lumen, followed by colonization of the large intestine by the trophozoites where they continue to divide and encyst. Eventually, both trophozoites and cysts are excreted in stools. Only 10% of the infected individuals will develop acute intestinal and extra-intestinal diseases. One possible explanation for this observation is the difference in the gut microbiota between individuals who may significantly influence the host’s immune response in amebiasis and *E. histolytica*’s virulence [1]. Over the last few decades, it has become evident that *E. histolytica*’s pathogenicity is directly linked to the parasite’s interaction with the gut microbiota [2], as the parasites are reported to feed on bacteria and cellular debris found in the large intestine [1]. However, such feeding is very selective, where only those bacteria with the appropriate recognition molecules are ingested by the parasite [3]. Amebiasis is characterized by acute inflammation of the intestine with release of pro-inflammatory cytokines, reactive oxygen species (ROS), and reactive nitrogen species (RNS) from activated cells of the host’s immune system. ROS and RNS are the major cytotoxic effectors for killing *E. histolytica* and cause oxidation and nitrosylation of amebic proteins, trigger stress responses, and inhibit glycolysis and the activity of some virulence factors [4,5,6,7]. Cellular means of subverting the toxicity of oxidative stress (OS) are important for the success of infectious diseases. No vaccine against amebiasis currently exists; the drug of choice for treating amebiasis is metronidazole, which may cause severe side effects such as nausea, vomiting, headaches, a metallic or bitter taste in the mouth, and more serious effects such as anorexia, ataxia, and skin rashes/itching [8,9]. Additionally, some clinical strains of *E. histolytica* are less sensitive to metronidazole, suggesting the emergence of metronidazole-resistant strains [10,11].

Probiotics are live organisms which, when administered in adequate amounts, confer a health benefit to the host [12,13]. Probiotics and commensal bacteria have been suggested to have some influence on the outcome of protozoan infections [14,15,16]. As an alternative bio-therapeutic for amebiasis, there are a number of studies which have been conducted, interestingly most of these studies are aimed at the efficiency of the probiotic at inhibiting adhesion of the protozoa to the intestinal mucosal surface [17,18]. Recently we have shown that *Lactobacillus acidophilus* is detrimental to *E. histolytica* [19]. This detrimental effect is associated with the transcription by the parasite of genes encoding major signaling molecules, such as kinases, regulators of small GTPases and oxidoreductases and genes encoding proteins necessary for ribosome structure. It has been suggested that the probiotic effect of certain bacteria (such as *L. acidophilus*) is mediated by the ability to produce H_2_O_2_ [20] via an NADH-dependent flavin reductase [21] and to maintain a normal, homeostatic microbiota [21]. In this study, we demonstrated that H_2_O_2_ produced by *L. acidophilus* caused the death of the parasite by oxidizing important amebic proteins. To our knowledge, this work provides the first comprehensive analysis of OXs in a protozoan parasite exposed to *L. acidophilus*.

## 2. Materials and Methods

### 2.1. E. histolytica and L. acidophilus Culture

*E. histolytica* trophozoites, the HM-1:IMSS strain (a gift from Samudrala Gourinath, Jawaharlal Nehru University, New Delhi, India), were grown and harvested according to a previously reported protocol [22].

*L. acidophilus* ATCC4356 strain was cultivated in De Man, Rogosa and Sharpe (MRS) media (Sigma-Aldrich, Jerusalem, Israel) overnight at 37 °C with agitation (200 rpm) on a New Brunswick Innova 4300 Incubator Shaker (Marshall Scientific, Hampton, NH, USA). Heat-killed *L. acidophilus* was cultivated in MRS media (Sigma-Aldrich, Jerusalem, Israel) overnight at 37 °C with agitation, followed by autoclaving at 121 °C and 1.05 kg/cm^2^ for 15 min.

### 2.2. Reagents

Catalase from bovine liver (C9322) was purchased from Sigma-Aldrich (Jerusalem, Israel).

### 2.3. Ferrous Oxidation-Xylenol Orange (FOX) Assay

The amount of H_2_O_2_ produced by *L. acidophilus* was determined by the FOX assay according to a previously reported protocol [23].

### 2.4. Viability of E. histolytica Trophozoites

Trophozoites (~1 × 10^6^/mL) were incubated with *L. acidophilus* (~1 × 10^9^/mL) in serum-free Diamond’s TYI S-33 medium for 120 min at 37 °C with agitation (200 rpm) in a thermoshaker (ALS-MS-100, Hangzhou Allsheng Instrument, Hangzhou, China). The viability of trophozoites was determined by the eosin dye exclusion method [6].

### 2.5. Detection of Oxidized Proteins (OXs) by Resin-Assisted Capture RAC (OX–RAC)

The detection of OXs by OX–RAC was performed on three biological replicates using a previously described protocol [6]. Captured proteins were eluted with 30-μL elution buffer containing 10 mM HEPES, 0.1 mM EDTA, 0.01 mM neocuproine, 0.1% sodium dodecyl sulfate (SDS), and 100 mM 2-mercaptoethanol for 20 min at room temperature. Proteins in a 10-μL aliquot of each eluent were resolved on a 12.5% SDS—polyacrylamide gel electrophoresis (PAGE) gel. Each gel was then stained with silver (Pierce Silver Stain), and each gel slice was independently analyzed by MS. A protein was considered to be oxidized when its relative amount in the DTT-treated lysates was significantly more than that in the DTT-untreated lysates (*p <* 0.05 according to the results of an unpaired *t*-test).

### 2.6. In-Gel Proteolysis and MS Analysis

The proteins in the gel were reduced with 2.8mM DTT (60 °C for 30 min), modified with 8.8 mM iodoacetamide in 100 mM ammonium bicarbonate (in the dark and at room temperature for 30 min) and digested in 10% acetonitrile and 10 mM ammonium bicarbonate with modified trypsin (Promega, Beit Haemek, Israel) overnight at 37 °C. A second trypsin digestion was carried out for another 4 h at 37 °C.

The tryptic peptides were desalted using C18 tips (Home-made, 3M) dried and re-suspended in 0.1% formic acid.

The peptides were resolved by reverse-phase chromatography on 0.075 X 180-mm fused silica capillaries (JW) packed with Reprosil reversed phase material (Dr Maisch GmbH, Germany). The peptides were eluted with linear 60 min gradient of 5 to 28% 15 min gradient of 28 to 95% and 25 min at 95% acetonitrile with 0.1% formic acid in water at flow rates of 0.15 µL/min. MS was performed by Q Exactive HF mass spectrometer (Thermo Fisher Scientific represented by BARGAL analytical instruments, Shoham, Israel) in a positive mode using a repetitively full MS scan followed by collision induces dissociation (HCD) of the 18 most dominant ions selected from the first MS scan. The mass spectrometry data were analyzed using the MaxQuant software 1.5.2.8, The Max Plank Institute of Biochemistry, Munich, Germany [24] vs. *Entamoeba histolytica* and *Lactobacillus acidophilus* proteomes from the Uniprot database with 1% FDR (false discovery rate). The data were quantified by label free analysis using the same software. Statistical analysis of the identification and quantization results was done using Perseus 1.6.7.0 software, The Max Plank Institute of Biochemistry, Munich, Germany [25]. A *t*-test between the groups with or without DTT was carried out, with the Benjamini–Hochberg correction for multiple testing. Proteins were considered as significantly changed if their *p*-value < 0.05, *q*-value < 0.05, and the fold change between the groups ≥1.

### 2.7. Classification of OXs According to Their Protein Class and Statistical Overrepresentation Test

The OXs were classified according to PANTHER Protein Class using the PANTHER Classification System software (http://www.pantherdb.org/, accessed on 28 July 2021) [26]. This classification of proteins derived from PANTHER/X molecular function ontology includes commonly used classes of protein families, many of which are not covered by GO molecular function.

Regarding the statistical overrepresentation test, the online system compares a list of genes of interest (in this work, genes encoding for OXs in *E. histolytica* trophozoites exposed to *L. acidophilus*) to a reference list (*E. histolytica* in database). The *p*-value calculation in the overrepresentation test is calculated automatically based on the number of genes expected in the test list for a particular PANTHER category, based on the reference list.

### 2.8. Measurement of Cysteine Proteases (CPs) Activity

CPs activity was monitored by cleavage of the synthetic substrate benzyloxycarbonyl-l-arginyl-l-arginine-p-nitroanilide (z-Arg-Arg-pNA) (Bachem, Torrance, CA, USA) using a previously described protocol [27] except that DTT was not systematically added to the reaction buffer. Briefly, z-Arg-Arg-pNA was incubated for 0–10 min at 37 °C with *E. histolytica* lysate (40 µg) (prepared in phosphate buffer saline (PBS) nonidet P-40 (1%) (Sigma-Aldrich, Israel) in 990 µL CP buffer (0.1 M KH2PO4, 2 mM EDTA, pH 7.0). Cleavage of Z-Arg-Arg-pNA substrate were detected at 405 nm in a Novaspec plus spectrophotometer (Sigma-Aldrich, Israel).

### 2.9. Adhesion Assay

The adhesion of *E. histolytica* trophozoites to HeLa cells (a kind gift from T. Kleinberger, Faculty of Medicine, Technion) was measured using a previously described protocol [28]. *E. histolytica* trophozoites (2 × 10^5^) were incubated with live *L. acidophilus* (2 × 10^8^), with heat-killed *L. acidophilus* (DN) (2 × 10^8^), with paraformaldehyde-fixed *L. acidophilus* (PLA) (2 × 10^8^) and with/without catalase (50 µg/mL) for 1 h at 37 °C and then transferred to paraformaldehyde-fixed HeLa cells monolayers for an additional hour of incubation at 37 °C. Trophozoites unattached to HeLa cell monolayers were washed once with phosphate buffer saline (PBS) buffer and the trophozoites attached to the HeLa cell monolayer were eluted with 500 µL of a solution of cold galactose (1%) in PBS and counted.

## 3. Results and Discussion

### 3.1. L. acidophilus Amebicide Activity Depends on the Formation of H_2_O_2_

**Figure 1:**
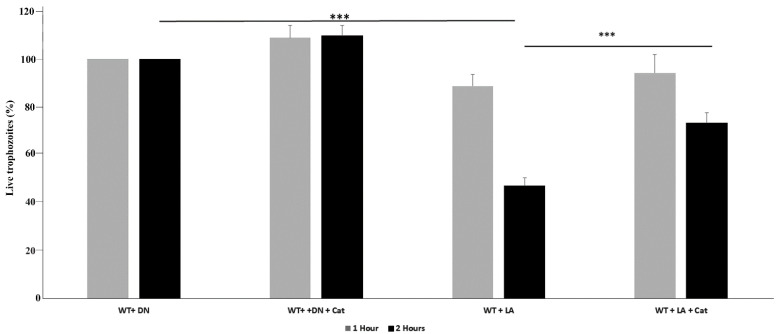
Viability assay of *E. histolytica* trophozoites. Note: *E. histolytica* trophozoites (WT) were incubated with live *L. acidophilus* (LA) or with heat-killed *L. acidophilus* (DN), with/without catalase (Cat) (50 µg/mL) for 60 and 120 min at 37 °C. The data represent two independent experiments performed in triplicate. *** *p* value < 0.001 by an unpaired Student’s *t*-test.

*L. acidophilus* is commonly found in the gastrointestinal tract of healthy humans. It is widely used as a food preservative and as a probiotic. *L. acidophilus* antimicrobial activity is caused by the production of antimicrobial peptides, including lactacins B, organic acid production such as lactic acids and H_2_O_2_ (recently reviewed in [29]), and immune induction [30]. Whereas the antibacterial and antifungal activity [31,32] of *L. acidophilus* has been well illustrated, the antiparasitic properties of *L. acidophilus* have been less studied. Studies with mouse models of the diseases caused by *Giardia lamblia* [33], *Toxocara canis* [34], *Trichinella spiralis* [35], and *Cryptosporidium parvum* [36] reveal that a combination of probiotics and other probiotic strains is beneficial in the treatment and prevention of these parasites. In a recent study, we demonstrated that *L. acidophilus* is detrimental to *E. histolytica* but the amebicide mechanism was unknown [19]. In this study, we investigated whether H_2_O_2_ generated by *L. acidophilus* is directly responsible for the amebicide activity. We first measured the ability of *L. acidophilus* to produce H_2_O_2_ by the FOX assay. We found that overnight culture of *L. acidophilus* cultivated in MRS media with agitation produces 0.14 ±0.3 mM H_2_O_2_. A viability assay was performed on *E. histolytica* trophozoites incubated either with *L. acidophilus* or with heat-killed *L. acidophilus*, which served as negative control. The viability of *E. histolytica* trophozoites was not affected when the parasite was incubated with *L. acidophilus* for 60 min (Figure 1). However, the viability of *E. histolytica* trophozoites was significantly decreased by 50% when the parasite was incubated with *L. acidophilus* for 120 min. In contrast, the viability of *E. histolytica* trophozoites incubated with heat-killed *L. acidophilus* for 120 min was not impaired (Figure 1). Next, we wanted to establish if the amebicidal activity of *L. acidophilus* was dependent on the formation of H_2_O_2_. We incubated *E. histolytica* and *L. acidophilus* in presence of catalase, an enzyme that catalyzes the decomposition of H_2_O_2_ to H_2_O and O_2_ [37]. We observed that the amebicidal activity of *L. acidophilus* was strongly reduced when catalase was added during the incubation of *L. acidophilus* with the parasite (Figure 1). Based on this finding, it strongly suggests that H_2_O_2_ produced by *L. acidophilus* is the primary cause of parasite death.

### 3.2. Resin-Assisted Capture (RAC) of Oxidized Proteins (OX) Coupled to Mass Spectrometry (OX–RAC) Analysis of E. histolytica Trophozoites Exposed to L. acidophilus

**Figure 2:**
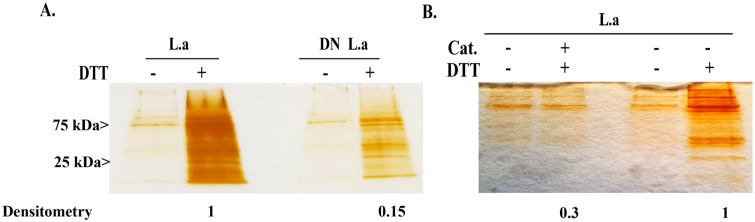
Detection of OXs by resin-assisted capture (OX–RAC) analysis of *E. histolytica.* Note: *E. histolytica* trophozoites were incubated with live *L. acidophilus* (L.a) or with heat-killed *L. acidophilus* (DN L.a) (**A**), with/without catalase (Cat.) (50 µg/mL) (**B**) for 2 h at 37 °C. Total protein lysate was prepared by lysing the trophozoites with 1% Igepal in PBS. The oxidized proteins in the cell lysates were subjected to RAC in the presence of 10 mM DTT (+DTT) or the absence of DTT (−DTT). The protein was resolved on a 12% SDS-PAGE and stained with silver stain.

In order to explore the amebicidal properties of *L. acidophilus*, we used OX–RAC to measure the levels of oxidized proteins (OXs) in *E. histolytica* trophozoites exposed to *L. acidophilus*. In absence of DTT treatment, OXs are not expected to bind to the thiopropyl resin [38]. We observed that the level of OXs in *E. histolytica* trophozoites exposed to heat-killed *L. acidophilus* culture is very low (Figure 2A). These results indicate that heat-killed culture of *L. acidophilus* do not trigger the formation of OXs in *E. histolytica* trophozoites. In contrast, a strong level of OXs was detected in *E. histolytica* trophozoites exposed to live *L. acidophilus* culture (Figure 2A). The addition of catalase during the interaction of *E. histolytica* trophozoites with *L. acidophilus* strongly inhibits the formation of OXs in the parasite, which confirms that the formation of OXs in the parasite is mediated by H_2_O_2_ produced by *L. acidophilus* (Figure 2B). These results indicate that the formation of OXs is triggered by H_2_O_2_ produced by *L. acidophilus*.

The intensity of the protein bands were quantified by densitometry using Image J software [39]. The intensity of the OX-protein bands obtained in the presence of DTT in *E. histolytica* trophozoites incubated with live *L. acidophilus* was arbitrarily set to 1. It is important to note that the data presented in Figure 2A,B were obtained at two different times, and that the silver staining development time was different in each case.

**Figure 3:**
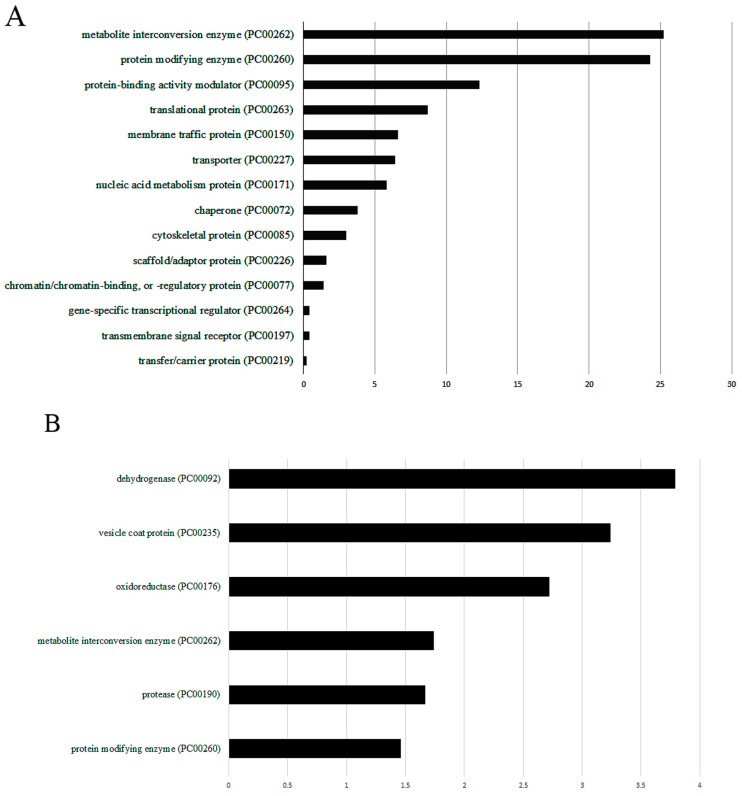
Protein analysis through evolutionary relationships (PANTHER) analysis of OXs in *E. histolytica* incubated with *L. acidophilus.* Note: (**A**) PANTHER sequence classification of the OXs identified in *E. histolytica* trophozoites co-incubated with *L. acidophilus*. (**B**) PANTHER statistical overrepresentation test of the OXs identified in *E. histolytica* trophozoites incubated with *L. acidophilus*.

Using MS, we identified 997 OXs in *E. histolytica* trophozoites incubated with *L. acidophilus* (Table S1), which were classified using PANTHER. The most abundant OX families belong to metabolite interconversion enzyme (PC00262), such as protein arginine N-methyltransferase (EHI_158560), the galactose-specific adhesin 170kD subunit (EHI_042370), and thioredoxin (EHI_004490) (Figure 3A). *E. histolytica* lacks glutathione, so it relies mainly on thiol for its defense against OS [40]. Thioredoxin (TRX)/thioredoxin reductase (TRXR) also contributes to redox signaling in *E. histolytica* trophozoites as well as oxidative stress responses [41]. This ubiquitous mechanism of defense is present in many parasites, including *Schistosoma mansoni*, *Plasmodium falciparum*, *Giardia lamblia*, and *Trichomonas vaginalis* [41]. TRXs are small redox proteins of around 12 kD, which act as radical scavengers. In their active site, two cysteine residues are involved in the antioxidant system. The oxidation of these cysteine residues produces disulfide bonds, which will be reduced by TRXR. The presence of TRXs as OXs in *E. histolytica* exposed to *L. acidophilus* strongly suggests that the parasite is actively responding to H_2_O_2_ released by the bacteria.

The other abundant OX family belongs to the protein modifying enzyme (PC00260) such as cysteine proteinase CP5 (EHI_168240), serine/threonine-protein phosphatase (EHI_031240), or E3 ubiquitin-protein ligase (EHI_050540) and the protein-binding activity modulator (PC00095) such as AIG1 family protein (EHI_176700), inhibitors of serine proteinase domain-containing protein (SERPIN) (EHI_119330), and the Rho family GTPase (EHI_070730) (Figure 3A).

SERPINs control a broad range of biological processes, including pathogen evasion of the host defense system. Cathepsin G, a pro-inflammatory enzyme released by activated neutrophils, is inhibited by serpins [42]. *E. histolytica* expresses a SERPIN that interacts with human neutrophil cathepsin G [43]. In this work, we showed that EhSERPIN is one of the OXs present in *E. histolytica* exposed to *L. acidophilus*. Studies have suggested that SERPINs are redox-regulated by oxidation of cysteine residues in the reactive site loop of these enzymes or its vicinity [44,45,46]. The presence of carbamidomethylated cysteine residues in the vicinity of the reactive site loop of EhSERPIN (Table S2) [43] suggests that EhSERPIN is also redox-regulated. The effect of oxidation on EhSERPIN activity has yet to be determined.

A functional motility is critical to the survival of *E. histolytica* in order to both dislodge and phagocytose host cells as well as transport virulence factors intracellularly [47]. Rho GTPases play a critical role in the regulation of motility and phagocytic activity of *E. histolytica* [48]. There are several Rho GTPases present in the parasite, and we identified six of them (EHI_126310, EHI_013260, EHI_197840, EHI_029020, EHI_129750, and EHI_070730) as OXs. EhRho1 (EHI_029020) regulates phagocytosis by regulating actin polymerization [49]. Numerous studies have shown that ROS regulate Rho GTPases activity [50]. Many Rho family GTPases contain a cysteine-containing motif (GXXXXGK[S/T]C) at their N-terminal, which is located directly adjacent to the phosphoryl-binding loop. Oxidation of the cysteine residue in this motif affects the nucleotide binding properties of these Rho GTPases [50]. According to the MS analysis of OXs (Table S2), this cysteine residue in the active site is not carbamidomethylated. Instead, we found that cysteine residues located at the C-terminal of these Rho GTPases are carbamidomethylated (Table S2). An ubiquitination region is present in the C-terminal region of many Rho GTPases that may regulate their stability [51]. In light of this information, it is tempting to speculate that the stability of these Rho GTPases is redox-dependent. An example of such regulation occurring in human endothelial cells is described here [52].

Of the OXs in *E. histolytica* trophozoites incubated with *L. acidophilus* (Table S2), oxidoreductase (PC00176) and dehydrogenase (PC00092), such as glyceraldehyde-3-phosphate dehydrogenase (EHI_008200), NAD(FAD)-dependent dehydrogenase (EHI_099700), and pyruvate: ferredoxin oxidoreductase (EHI_051060), are significantly enriched according to the PANTHER statistical overrepresentation test (Figure 3B). Pyruvate: ferredoxin oxidoreductase (EHI_051060) is a Fe–S enzyme that catalyzes the oxidative decarboxylation of pyruvate [53]. This protein has also been identified as an OX in trophozoites exposed to H_2_O_2_ [6], metronidazole, or auranofin [54]. In an oxidatively stressed parasite, pyruvate: ferredoxin oxidoreductase becomes strongly inhibited, resulting in an accumulation of pyruvate, which limits ATP production and causes parasite death [55]. Several cysteine residues present within the [4Fe–4S] clusters of close to them are carbamidomethylated suggesting that they are oxidized (Table S2). Destabilization of the Fe–S clusters integrity via oxidation of these cysteine residues in the parasite exposed to *L. acidophilus* will more certainly inactivate the enzyme and consequently contribute to the parasite death.

Other OXs, which are significantly enriched according to the PANTHER statistical overrepresentation test, include vesicle coat protein (PC00235), such as GOLD domain-containing protein (EHI_023070), beta2-COP (EHI_088220) and coatomer subunit gamma (EHI_040700) and protease (PC00190), such as EhCP-a1 (EHI_074180) and EhCP-a4 (EHI_050570) (Figure 3B).

### 3.3. E. histolytica CP Activity Is Impaired by L. acidophilus

**Figure 4:**
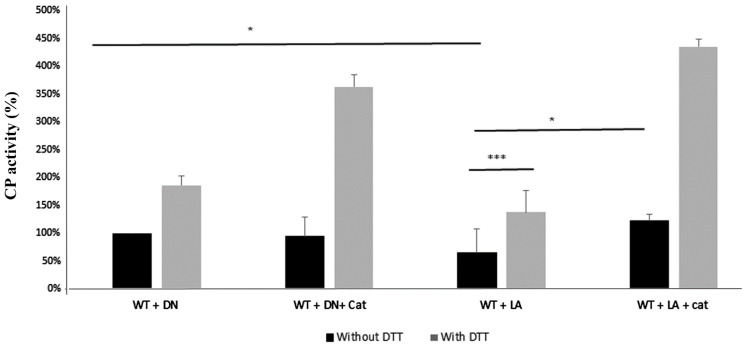
CPs activity of *E. histolytica* trophozoites. Note: *E. histolytica* trophozoites were incubated with heat-killed *L. acidophilus* (DN) or with live *L. acidophilus* (LA), and with/without catalase (50 µg/mL) for 2 h at 37 °C. Total protein was prepared and CPs activity was measured. One unit of CP activity was defined as the number of micromoles of substrate digested per minute per milligram of protein. CP activity performed without DTT of *E. histolytica* trophozoites incubated with heat-killed *L. acidophilus* (WT + DN) was obtained as 100% and it corresponds to 0.31 units. The data represent two independent experiments performed in triplicate. * *p*-value < 0.05 by an unpaired Student’s *t*-test. *** *p*-value < 0.001 by an unpaired Student’s *t*-test.

In order to gain information on the consequence of *L. acidophilus*-mediated-oxidation on the activity of proteins that were identified in the OX–RAC analysis, we decided to focus here on the CPs. When trophozoites are incubated with live *L. acidophilus*, CPs activity is strongly inhibited (Figure 4). However, this activity is not inhibited when trophozoites are incubated with *L. acidophilus* in the presence of catalase (Figure 4). The addition of DTT in lysates of trophozoites incubated with live *L. acidophilus* partially restored CP activity. Based on these results, it could be assumed that the *L. acidophilus*-mediated-oxidation of CPs’ catalytic cysteine residues inhibits CPs, while their reduction by DTT restores the activity. Indeed, the fact that adding catalase to trophozoites incubated with *L. acidophilus* prevents the inhibition of CPs confirms that H_2_O_2_ produced by *L. acidophilus* inhibits the CPs.

CPs are essential for the growth of *E. histolytica* trophozoites and their inhibition by inhibitors of the CPS, such as E64d, causes their death [56]. In this study, we found that many CPs, including EhCP-a1 (EHI_074180), EhCP-a4 (EHI_050570), EhCP-a5 (EHI_168240), and EhCP8 (EHI_010850), are oxidized, and that *E. histolytica* CPs activity are inhibited when the parasite is incubated with *L. acidophilus*. Some of these OXs CPs, such as EhCP-A1 and EhCP-A5, are highly expressed in *E. histolytica* [57] and are involved in rosette formation, hemolysis, and erythrocyte digestion [58]. The expression of EHI_010850 (EhCP-8) is upregulated when the parasite is incubated in the presence of hemoglobin, which suggests CP-8 is involved in iron uptake by the parasite [59]. The mechanisms that lead to oxidants inhibiting CPs have recently been examined [60]. For example, inhibition of papain by H_2_O_2_ results from the formation of sulfenic acid, which reacts with adjacent free thiol to form mixed disulfides. In addition, H_2_O_2_ inhibits cathepsin B by targeting the active site residue (Cys25) to form either sulfenic acid or sulfonic acid around 70% of the time. *E. histolytica* CPs contain four active-site residues, namely Gln, Cys, His, and Asn, the cysteine residue at the active site being present in all *E. histolytica* CPs [61]. According to the MS analysis of OXs (Table S2), this cysteine residue in the active site is carbamidomethylated, which strongly suggests that it was oxidized. By itself, this observation would explain why *E. histolytica*’s CP activity is inhibited by H_2_O_2_ produced by *L. acidophilus*. As opposed to *E. histolytica*, where H_2_O_2_ produced by *L. acidophilus* appears to inhibit CPs activity directly, in *Plasmodium* parasites, H_2_O_2_-mediated inhibition of CPs is dependent on the presence of free hemin, which can be released by quinoline drugs [62].

### 3.4. Adhesion of E. histolytica Trophozoites to HeLa Cells Is Impaired by L. acidophilus

**Figure 5:**
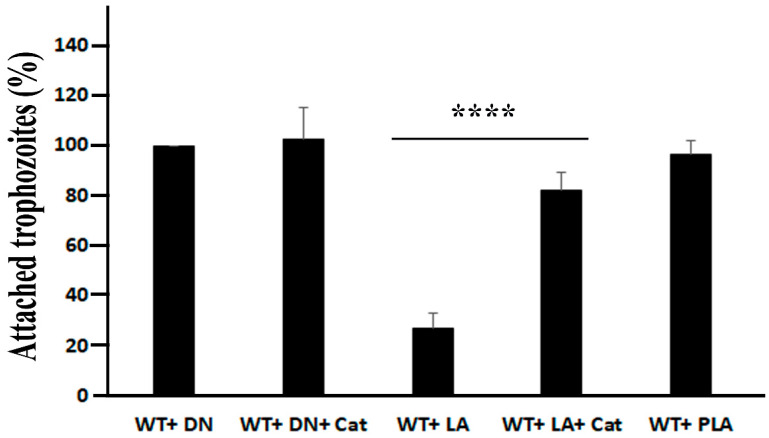
Binding activity assay of *E. histolytica* trophozoites. Note: *E. histolytica* trophozoites were incubated with live *L. acidophilus* (LA), with heat-killed *L. acidophilus* (DN), with paraformaldehyde-fixed *L. acidophilus* (PLA), and with/without catalase (50 µg/mL) for 1 h at 37 °C and then transferred to paraformaldehyde-fixed HeLa cell monolayers. Trophozoites attached to HeLa cells monolayers were counted. The number of trophozoites incubated with heat-killed *L. acidophilus* (WT + DN) that were bound to HeLa cells monolayer (around 75% of the original population) was obtained as 100%. The data represent two independent experiments performed in duplicate. **** *p*-value < 0.0001 by an unpaired Student’s *t*-test.

*E. histolytica* trophozoites’ ability to bind to mammalian cells is the initial step in the amebic infectious process [63]. In our experiment, trophozoites incubated with *L. acidophilus* exhibit reduced binding to HeLa cells compared to trophozoites incubated with heat-killed *L. acidophilus* or with paraformaldehyde-fixed *L. acidophilus*. However, the binding activity to HeLa cells of trophozoites incubated with *L. acidophilus* in the presence of catalase is comparable to the binding activity of heat-killed *L. acidophilus* or with paraformaldehyde-fixed *L. acidophilus* (Figure 5). These data strongly suggest that the production of H_2_O_2_ by *L. acidophilus* inhibits *E. histolytica*’s binding to HeLa cells rather than a competition between *L. acidophilus* and HeLa cells. The lectin Gal/GalNAc plays an essential role in parasite attachment to mammalian cells, including HeLa cells [64,65,66]. We previously demonstrated that oxidation of the carbohydrate-recognizing cysteine-rich domain (CRD) of Gal/GalNAc lectin renders it inactive [6]. We observed in this study that 170kDa Gal/GalNAc is one of the OXs produced in the parasite exposed to *L. acidophilus*. According to the MS analysis of OXs (Table S2), many cysteine residues are carbamidomethylated in the CRD of Gal/GalNAc lectin, which strongly suggests that they were oxidized leading to an impairment of the parasite’s ability to bind mammalian cells (this work and [6]).

## 4. Conclusions

The results for this study show that the production of H_2_O_2_ by *L. acidophilus* causes oxidation of vital proteins in *E. histolytica* and ultimately results in parasite death. The present study emphasizes *L. acidophilus*’ potential as a probiotic against amebiasis. However, in vivo trials are necessary to determine whether this probiotic has health benefits on humans when it is used alone or in combination with metronidazole.

## Data Availability

Data is contained within the article and Supplementary Material.

## Supplementary Materials

The following are available online at https://www.mdpi.com/article/10.3390/antiox11050814/s1. Table S1: List of all OXs that were enriched by RAC in three independent experiments in *E. histolytica* trophozoites incubated with *L. acidophilus*. Table S2: List carbamidomethyl (C) sites in OXs. Table S3: Description of the parameters that are given in Table S1 and Table S2.

## References

[1] Marie C., Petri W.A. (2014). Regulation of Virulence of *Entamoeba* histolytica. Annu. Rev. Microbiol..

[2] Gill N.J., Ganguly N.K., Mahajan R.C., Dilawari J.B. (1988). Antibody dependent cellular cytotoxicity in experimental intestinal & hepatic amoebiasis. Indian J. Med. Res..

[3] Schulz T.F., Kollaritsch H., Hengster P., Stemberger H., Scheiner O., Wiedemann G., Dierich M.P. (1987). Molecular weight analysis of *Entamoeba* histolytica antigens recognized by IgG and IgM antibodies in the sera of patients with amoebiasis. Trop. Med. Parasitol..

[4] Rastew E., Vicente J.B., Singh U. (2012). Oxidative stress resistance genes contribute to the pathogenic potential of the anaerobic protozoan parasite, *Entamoeba* histolytica. Int. J. Parasitol..

[5] Santi-Rocca J., Smith S., Weber C., Pineda E., Hon C.-C., Saavedra E., Olivos-García A., Rousseau S., Dillies M.-A., Coppée J.-Y. (2012). Endoplasmic reticulum stress-sensing mechanism is activated in *Entamoeba* histolytica upon treatment with nitric oxide. PLoS ONE.

[6] Shahi P., Trebicz-Geffen M., Nagaraja S., Alterzon S.B., Hertz R., Methling K., Lalk M., Ankri S. (2016). Proteomic Identification of Oxidized Proteins in *Entamoeba* histolytica by Resin-Assisted Capture: Insights into the Role of Arginase in Resistance to Oxidative Stress. PLoS Negl. Trop. Dis..

[7] Hertz R., Ben Lulu S., Shahi P., Trebicz-Geffen M., Benhar M., Ankri S. (2014). Proteomic Identification of S-Nitrosylated Proteins in the Parasite *Entamoeba histolytica* by Resin-Assisted Capture: Insights into the Regulation of the Gal/GalNAc Lectin by Nitric Oxide. PLoS ONE..

[8] Roe F.J. (1977). Metronidazole: Review of uses and toxicity. J. Antimicrob. Chemother..

[9] Andersson K.E. (1981). Pharmacokinetics of Nitroimidazoles—Spectrum of Adverse Reactions. Scand. J. Infect. Dis..

[10] Bansal D., Sehgal R., Chawla Y., Mahajan R.C., Malla N. (2004). In vitro activity of antiamoebic drugs against clinical isolates of *Entamoeba histolytica* and *Entamoeba* dispar. Ann. Clin. Microbiol. Antimicrob..

[11] Iyer L.R.B.N., Naik S., Paul J. (2017). Antioxidant enzyme profile of two clinical isolates of *Entamoeba histolytica* varying in sensitivity to antiamoebic drugs. World J. Clin. Infect. Dis..

[12] Hill C., Guarner F., Reid G., Gibson G.R., Merenstein D.J., Pot B., Morelli L., Canani R.B., Flint H.J., Salminen S. (2014). Expert consensus document. The International Scientific Association for Probiotics and Prebiotics consensus statement on the scope and appropriate use of the term probiotic. Nat. Rev. Gastroenterol. Hepatol..

[13] Travers M.A., Florent I., Kohl L., Grellier P. (2011). Probiotics for the control of parasites: An overview. J. Parasitol. Res..

[14] Bar A.K., Phukan N., Pinheiro J., Simoes-Barbosa A. (2015). The Interplay of Host Microbiota and Parasitic Protozoans at Mucosal Interfaces: Implications for the Outcomes of Infections and Diseases. PLoS Negl. Trop. Dis..

[15] Goyal N., Tiwari R.P., Shukla G. (2011). Lactobacillus rhamnosus GG as an Effective Probiotic for Murine Giardiasis. Interdiscip. Perspect. Infect. Dis..

[16] Sarjapuram N., Mekala N., Singh M., Tatu U. (2017). The Potential of Lactobacillus casei and Entercoccus faecium Combination as a Preventive Probiotic Against *Entamoeba*. Probiotics Antimicrob. Proteins.

[17] Rigothier M.C., Maccario J., Gayral P. (1994). Inhibitory activity of saccharomyces yeasts on the adhesion of *Entamoeba histolytica* trophozoites to human erythrocytes in vitro. Parasitol. Res..

[18] Mansour-Ghanaei F., Dehbashi N., Yazdanparast K., Shafaghi A. (2003). Efficacy of saccharomyces boulardii with antibiotics in acute amoebiasis. World J. Gastroenterol..

[19] Varet H., Shaulov Y., Sismeiro O., Trebicz-Geffen M., Legendre R., Coppée J.-Y., Ankri S., Guillen N. (2018). Enteric bacteria boost defences against oxidative stress in *Entamoeba histolytica*. Sci. Rep..

[20] Collins E.B., Aramaki K. (1980). Production of Hydrogen peroxide by Lactobacillus acidophilus. J. Dairy Sci..

[21] Hertzberger R., Arents J., Dekker H.L., Pridmore R.D., Gysler C., Kleerebezem M., de Mattos M.J.T. (2014). H_2_O_2_ production in species of the Lactobacillus acidophilus group: A central role for a novel NADH-dependent flavin reductase. Appl. Environ. Microbiol..

[22] Diamond L.S., Harlow D.R., Cunnick C.C. (1978). A new medium for the axenic cultivation of *Entamoeba histolytica* and other *Entamoeba*. Trans. R. Soc. Trop. Med. Hyg..

[23] DeLong J.M., Prange R.K., Hodges D.M., Forney C., Bishop M.C., Quilliam M. (2002). Using a modified ferrous oxidation-xylenol orange (FOX) assay for detection of lipid hydroperoxides in plant tissue. J. Agric. Food Chem..

[24] Cox J., Hein M.Y., Luber C.A., Paron I., Nagaraj N., Mann M. (2014). Accurate proteome-wide label-free quantification by delayed normalization and maximal peptide ratio extraction, termed MaxLFQ. Mol. Cell. Proteom..

[25] Tyanova S., Temu T., Sinitcyn P., Carlson A., Hein M.Y., Geiger T., Mann M., Cox J. (2016). The Perseus computational platform for comprehensive analysis of (prote)omics data. Nat. Methods.

[26] Mi H., Ebert D., Muruganujan A., Mills C., Albou L.-P., Mushayamaha T., Thomas P.D. (2021). PANTHER version. 16, a revised family classification, tree-based classification tool, enhancer regions and extensive API. Nucleic Acids Res..

[27] Ankri S., Stolarsky T., Mirelman D. (1998). Antisense inhibition of expression of cysteine proteinases does not affect *Entamoeba histolytica* cytopathic or haemolytic activity but inhibits phagocytosis. Mol. Microbiol..

[28] Shahi P., Trebicz-Geffen M., Nagaraja S., Hertz R., Alterzon-Baumel S., Methling K., Lalk M., Mazumder M., Samudrala G., Ankri S. (2016). N-acetyl ornithine deacetylase is a moonlighting protein and is involved in the adaptation of *Entamoeba histolytica* to nitrosative stress. Sci. Rep..

[29] Dinev T.B.G., Denev S., Dermendzhieva D., Tzanova M., Valkova E. (2017). Antimicrobial activity of Lactobacillus acidophilus against pathogenic and food spoilage microorganisms: A review. Agric. Sci. Technol..

[30] Weiss G., Rasmussen S., Zeuthen L.H., Nielsen B.N., Jarmer H., Jespersen L., Frøkiaer H. (2010). Lactobacillus acidophilus induces virus immune defence genes in murine dendritic cells by a Toll-like receptor-2-dependent mechanism. Immunology.

[31] Salari S., Ghasemi Nejad Almani P. (2020). Antifungal effects of Lactobacillus acidophilus and Lactobacillus plantarum against different oral Candida species isolated from HIV/AIDS patients: An in vitro study. J. Oral Microbiol..

[32] Aween M.M., Hassan Z., Muhialdin B.J., Eljamel Y.A., Al-Mabrok A.S.W., Lani M.N. (2012). Antibacterial activity of Lactobacillus acidophilus strains isolated from honey marketed in Malaysia against selected multiple antibiotic resistant (MAR) Gram-positive bacteria. J. Food Sci..

[33] Al-Megrin W.A., Mohamed S.H., Saleh M.M., Yehia H.M. (2021). Preventive role of probiotic bacteria against gastrointestinal diseases in mice caused by Giardia lamblia. Biosci. Rep..

[34] Cadore P.S., Walcher D.L., de Sousa N.F.G.C., Martins L.H.R., da Hora V.P., Von Groll A., de Moura M.Q., Berne M.E.A., Avila L.F.D.C.D., Scaini C.J. (2021). Protective effect of the probiotic Lactobacillus acidophilus ATCC. 4356 in BALB/c mice infected with Toxocara canis. Rev. Inst. Med. Trop. São Paulo.

[35] Farrag H.M., Huseein E.A., Abd El-Rady N.M., Mostafa F.A., Mohamed S.S., Gaber M. (2021). The protective effect of Lactobacillus acidophilus on experimental animals challenged with Trichinella spiralis; new insights on their feasibility as prophylaxis in Trichinella spiralis endemic area. Ann. Parasitol..

[36] Alak J.I., Wolf B.W., Mdurvwa E.G., EPimentel-Smith G., Kolavala S., Abdelrahman H., Suppiramaniam V. (1999). Supplementation with Lactobacillus reuteri or *L. acidophilus* reduced intestinal shedding of cryptosporidium parvum oocysts in immunodeficient C57BL/6 mice. Cell. Mol. Biol. (Noisy-le-grand).

[37] Loew O. (1900). A New Enzyme of General Occurrence in Organismis. Science.

[38] Shaulov Y., Sarid L., Trebicz-Geffen M., Ankri S. (2021). *Entamoeba histolytica* Adaption to Auranofin: A Phenotypic and Multi-Omics Characterization. Antioxidants.

[39] Rueden C.T., Schindelin J., Hiner M.C., Dezonia B.E., Walter A.E., Arena E.T., Eliceiri K.W. (2017). ImageJ2: ImageJ for the next generation of scientific image data. BMC Bioinform..

[40] Fahey R.C., Newton G.L., Arrick B., Overdank-Bogart T., Aley S.B. (1984). *Entamoeba histolytica*: A eukaryote without glutathione metabolism. Science.

[41] Jeelani G., Nozaki T. (2016). *Entamoeba* thiol-based redox metabolism: A potential target for drug development. Mol. Biochem. Parasitol..

[42] Bao J., Pan G., Poncz M., Wei J., Ran M., Zhou Z. (2018). Serpin functions in host-pathogen interactions. PeerJ.

[43] Riahi Y., Siman-Tov R., Ankri S. (2004). Molecular cloning, expression and characterization of a serine proteinase inhibitor gene from *Entamoeba histolytica*. Mol. Biochem. Parasitol..

[44] Morris E.C., Dafforn T.R., Forsyth S.L., Missen M.A., Horvath A.J., Hampson L., Hampson I.N., Currie G., Carrell R.W., Coughlin P.B. (2003). Murine serpin. 2A is a redox-sensitive intracellular protein. Biochem. J..

[45] Stief T.W., Aab A., Heimburger N. (1988). Oxidative inactivation of purified human alpha-2-antiplasmin, antithrombin III, and C1-inhibitor. Thromb. Res..

[46] Mangan M.S., Bird C.H., Kaiserman D., Matthews A.Y., Hitchen C., Steer D.L., Thompson P.E., Bird P.I. (2016). A Novel Serpin Regulatory Mechanism: SerpinB9 Is Reversibly Inhibited by vicinal disulfide bond formation in the reactive center loop. J. Biol. Chem..

[47] Coudrier E., Amblard F., Zimmer C., Roux P., Olivo--Marin J.C., Rigothier M.C., Guillén N. (2005). Myosin II and the Gal-GalNAc lectin play a crucial role in tissue invasion by *Entamoeba histolytica*. Cell. Microbiol..

[48] Bosch D.E., Siderovski D.P. (2013). G protein signaling in the parasite *Entamoeba histolytica*. Exp. Mol. Med..

[49] Bharadwaj R., Sharma S., Janhawi Arya R., Bhattacharya S., Bhattacharya A. (2018). EhRho1 regulates phagocytosis by modulating actin dynamics through EhFormin1 and EhProfilin1 in *Entamoeba histolytica*. Cell. Microbiol..

[50] Hobbs G.A., Zhou B., Cox A.D., Campbell S.L. (2014). Rho GTPases, oxidation, and cell redox control. Small GTPases.

[51] Olson M.F. (2018). Rho GTPases, their post-translational modifications, disease-associated mutations and pharmacological inhibitors. Small GTPases.

[52] Kovacic H.N., Irani K., Goldschmidt-Clermont P.J. (2001). Redox regulation of human Rac1 stability by the proteasome in human aortic endothelial cells. J. Biol. Chem..

[53] Reeves R.E., Warren L.G., Susskind B., Lo H.S. (1977). An energy-conserving pyruvate-to-acetate pathway in *Entamoeba histolytica*. Pyruvate synthase and a new acetate thiokinase. J. Biol. Chem..

[54] Shaulov Y., Nagaraja S., Sarid L., Trebicz-Geffen M., Ankri S. (2020). Formation of oxidised (OX) proteins in *Entamoeba histolytica* exposed to auranofin and consequences on the parasite virulence. Cell. Microbiol..

[55] Pineda E., Encalada R., Rodríguez-Zavala J.S., Olivos-García A., Moreno-Sánchez R., Saavedra E. (2010). Pyruvate:ferredoxin oxidoreductase and bifunctional aldehyde-alcohol dehydrogenase are essential for energy metabolism under oxidative stress in *Entamoeba histolytica*. FEBS J..

[56] Nowak N., Lotter H., Tannich E., Bruchhaus I. (2004). Resistance of *Entamoeba histolytica* to the cysteine proteinase inhibitor E64 is associated with secretion of pro-enzymes and reduced pathogenicity. J. Biol. Chem..

[57] Matthiesen J., Bär A.-K., Bartels A.-K., Marien D., Ofori S., Biller L., Tannich E., Lotter H., Bruchhaus I. (2013). Overexpression of specific cysteine peptidases confers pathogenicity to a nonpathogenic *Entamoeba histolytica* clone. mBio.

[58] Irmer H., Tillack M., Biller L., Handal G., Leippe M., Roeder T., Tannich E., Bruchhaus I. (2009). Major cysteine peptidases of *Entamoeba histolytica* are required for aggregation and digestion of erythrocytes but are dispensable for phagocytosis and cytopathogenicity. Mol. Microbiol..

[59] Hernandez-Cuevas N.A., Weber C., Hon C.C., Guillen N. (2014). Gene expression profiling in *Entamoeba histolytica* identifies key components in iron uptake and metabolism. PLoS ONE.

[60] Lalmanach G., Saidi A., Bigot P., Chazeirat T., Lecaille F., Wartenberg M. (2020). Regulation of the Proteolytic Activity of Cysteine Cathepsins by Oxidants. Int. J. Mol. Sci..

[61] Bruchhaus I., Loftus B.J., Hall N., Tannich E. (2003). The intestinal protozoan parasite *Entamoeba histolytica* contains. 20 cysteine protease genes, of which only a small subset is expressed during in vitro cultivation. Eukaryot. Cell.

[62] Herraiz T., Guillen H., Gonzalez-Pena D., Aran V.J. (2019). Antimalarial Quinoline Drugs Inhibit beta-Hematin and Increase Free Hemin Catalyzing Peroxidative Reactions and Inhibition of Cysteine Proteases. Sci. Rep..

[63] Mirelman D., Kobiler D. (1981). Adhesion properties of *Entamoeba histolytica*. Adhesion and Microorganism Pathogenicity.

[64] Aguirre Garcia M., Gutierrez-Kobeh L., Lopez Vancell R. (2015). *Entamoeba histolytica*: Adhesins and lectins in the trophozoite surface. Molecules.

[65] Petri W.A., Haque R., Mann B.J. (2002). The bittersweet interface of parasite and host: Lectin-carbohydrate interactions during human invasion by the parasite *Entamoeba histolytica*. Annu. Rev. Microbiol..

[66] Vines R.R., Ramakrishnan G., Rogers J.B., Lockhart L.A., Mann B.J., Petri W.A. (1998). Regulation of adherence and virulence by the *Entamoeba histolytica* lectin cytoplasmic domain, which contains a beta2 integrin motif. Mol. Biol. Cell.

